# Vasomotor symptoms in midlife women with incident breast cancer: pink SWAN

**DOI:** 10.1007/s10549-021-06425-y

**Published:** 2021-10-25

**Authors:** Ellen B. Gold, Sybil L. Crawford, Katherine Leung, Gail Greendale, Katherine W. Reeves, Hadine Joffe, Nancy E. Avis

**Affiliations:** 1 University of California Davis School of Medicine, Davis, USA; 2 University of Massachusetts Medical School, Worcester, USA; 3 David Geffen School of Medicine, University of California Los Angeles, Los Angeles, USA; 4 University of Massachusetts Amherst School of Public Health and Health Sciences, Amherst, USA; 5 Harvard Medical School, Brigham and Women’s Hospital, Boston, USA; 6 Wake Forest University School of Medicine, Winston-Salem, USA; 7 Department of Public Health Sciences, University of California Davis, One Shields Ave., Med Sci 1C, Davis, CA 95616 USA

**Keywords:** Breast cancer, Vasomotor symptoms, Risk factors, Menopause

## Abstract

**Purpose:**

We compared trajectories of vasomotor symptoms (VMS) and their risk factors in women with breast cancer (BrCa) to those of cancer-free controls.

**Methods:**

Data were from 15 nearly annual follow-up visits (1996–2017) of the multi-racial/ethnic cohort of midlife women enrolled in the Study of Women’s Health Across the Nation (SWAN). We compared women with incident BrCa to controls for patterns of VMS, controlling for risk factors identified in bivariate analyses using multivariable longitudinal analyses.

**Results:**

Characteristics at study entry largely did not differ between cases (*n* = 151) and controls (*n* = 2161). Adjusted *prevalence* of *any* VMS increased significantly among cases from diagnosis to 2.75 years post diagnosis [per-year adjusted odds ratio (aOR) = 1.76, 95% confidence interval (CI) 1.39–2.24], peaking at 2.75 years post diagnosis, whereas prevalence was stable among controls in this interval [aOR = 1.04, 95% CI 0.99–1.11]. Beyond 2.75 years post diagnosis, *prevalence* declined significantly in cases [aOR = 0.72, 95% CI 0.61–0.84] and less in controls [aOR = 0.96, 95% CI 0.92–1.00]. Patterns were similar for *frequent* VMS. Adjustment for tamoxifen use slightly reduced the per-year OR for *any prevalent* VMS post diagnosis, partially explaining excess VMS in cases. Other treatments were unassociated with VMS.

**Conclusions:**

Patterns of prevalent VMS reporting differed significantly between cases and controls, particularly post diagnosis, the latter only partially explained by tamoxifen use among cases. Risk factors for VMS largely did not differ between cases and controls.

## Introduction

Breast cancer (BrCa) is the most frequently occurring cancer among US women. Currently, about 3.8 million BrCa survivors are alive in the US (https://www.bcrf.org/breast-cancer-statistics-and-resources), and 89.9% of the estimated 268,600 women diagnosed with BrCa annually will be alive 5 years after diagnosis (https://seer.cancer.gov/statfacts/html/breast.html), a number projected to increase. Vasomotor symptoms (VMS) are the most frequent symptoms for women undergoing the menopausal transition (MT) [1, 2]. Greater prevalence [3], frequency (6 vs. 3.1 per 24 h [4]), and severely troubling VMS (quite or extremely bothered 55.4% vs. 12.1% [5]) have been reported by a greater proportion of women treated for BrCa than among those without a BrCa history, particularly among those taking aromatase inhibitors (AIs) [6, 7] or tamoxifen [8] or experiencing treatment-induced menopause [9, 10]. VMS can adversely affect sleep [9], quality of life [10, 11], depressive symptoms [12] and adherence to BrCa treatment [13–15], which may affect survival [16–18], although those with AI-associated VMS have improved disease-free survival (adjusted hazard ratio [aHR] 0.47) [19].

The Australian Longitudinal Study on Women’s Health showed no association of VMS with incident BrCa (aHR 1.09) [20]. In the Life and Longevity After Cancer study, post-diagnosis VMS were significantly associated with chemotherapy (aOR 1.80), adjuvant hormone therapy (aOR 2.73), prior VMS (aOR 2.20), and older age [21]. The Study of Women’s Health Across the Nation (SWAN) found VMS were protective for BrCa incidence (aHR 0.63) during 11.4 years of follow-up [22]. Here, we extended follow-up to 15 SWAN visits and investigated VMS pre- and post-diagnosis to elucidate whether VMS patterns differed between women with and without BrCa or reflected BrCa treatment effects. Additionally, we compared risk factors for VMS in women with BrCa to those without cancer. We addressed the following hypotheses:

1. among women diagnosed with BrCa during follow-up, incidence and prevalence rates of VMS would be higher after diagnosis and cancer treatment than before;
2. prevalence rates of VMS in BrCa cases would be lower than in controls pre-diagnosis but higher post diagnosis;
3. in cases, treatment with BrCa-related selective estrogen receptor modulators (BrCa SERMS), endocrine medications, chemotherapy, or radiotherapy would be associated with greater prevalent VMS post treatment; and.
4. risk factors for VMS pre- and post-diagnosis among BrCa cases would not differ from controls and those found previously in SWAN [2].

## Methods

### Study participants

SWAN is a seven-site, longitudinal study of a multi-racial/ethnic cohort, characterizing physiological and psychosocial changes during the MT and assessing their relations to subsequent health. Each site recruited community-dwelling midlife women using random–digit dialing and/or list-based sampling [23] to identify Caucasians and one minority sample (African Americans in Boston, Pittsburgh, Chicago and Detroit; Hispanics in Newark, New Jersey; Japanese in Los Angeles; and Chinese in Oakland, CA). SWAN’s cross-sectional survey screened women for eligibility for the longitudinal study. Eligibility for the cross-sectional study included: resident in the geographic area of one of the sites, age 40–55 years, one of the target racial/ethnic groups, and spoke English or Japanese in Los Angeles, Spanish in New Jersey, or Cantonese in Oakland. Cohort eligibility included: aged 42–52 years, at least one menstrual period in the prior three months, not pregnant or lactating and not using exogenous sex steroids. Of 16,065 cross-sectional participants, 6521 were cohort eligible, and 3302 (50.6%) enrolled.

**Fig. 1:**
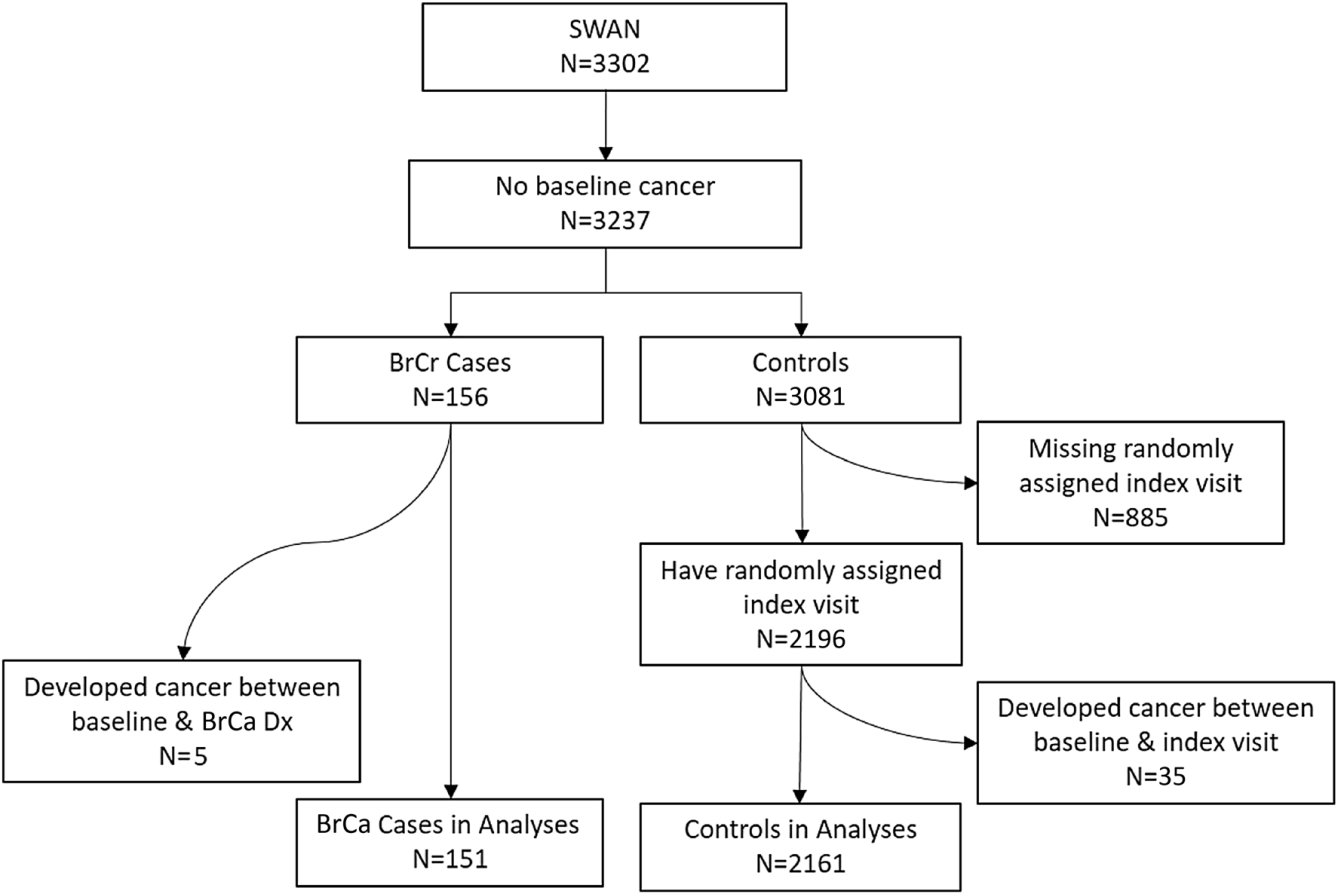
Flowchart for derivation of case and control samples

For this study (Pink SWAN), *cases* were women who developed BrCa since SWAN’s enrollment and had no cancer (except non-melanoma skin cancer) prior to developing BrCa. *Controls* comprised women with no cancer (other than non-melanoma skin cancer) prior to their index visit (defined below). Data for cases were censored after any recurrence or other cancer that developed after their BrCa diagnosis and for controls after any cancer developed after their index visit. Over 15 follow-up visits, we identified 151 incident BrCa cases and 2161 controls (Fig. 1). BrCa risk status was not ascertained for controls.

Adjudication of participant medical records began at visit 12. Of 109 cases for whom medical records were received, 103 cases were confirmed (94.5% agreement with self-report). For each case, we ascertained the date of diagnosis. To assign a corresponding date in controls, an index date was randomly assigned, using frequency matching to cases for first post-diagnosis visit, and randomly assigning a date between the last pre-diagnosis visit and the first post-diagnosis visit, such that the distributions of diagnosis/index dates were comparable in cases and controls.

Exclusions were: BrCa diagnosed prior to baseline (*N* = 28); other cancer diagnosed prior to baseline (*N* = 37) or between baseline and diagnosis/index visit (*N* = 104); and potential controls who missed a study visit corresponding to their randomly assigned index date (*N* = 885) (Fig. 1). Of these 885, 716 did not have at least one visit prior to and one visit after the index visit, so that interpolating data for a visit was not feasible. Compared with included controls, those excluded were more likely to be from the New Jersey site and Hispanic, reflecting a hiatus in data collection for visits 6–8 and 10–11 at that site. The higher proportion of Hispanics likely explains the slightly younger age, higher body mass index (BMI), lower educational attainment, higher anxiety and depressive symptom scores, and greater proportion of smokers among those excluded. Multivariate analyses adjusted for these characteristics.

The study protocol common to all sites was approved by each site’s institutional review board, and all participants provided written, signed informed consent.

### Data collection

#### Procedures

Participants were followed with approximately annual in-person visits from 1996 to 2017. All visits included: consent; a standard protocol for measuring height with a stadiometer and weight with a balance beam scale; and interviewer- and self-administered questionnaires, including questions on demographic characteristics, medical and reproductive history, medications, psychosocial measures, and symptoms. Questionnaires were forward- and back-translated into Spanish, Cantonese, and Japanese and administered by bilingual interviewers to participants preferring these languages.

#### Outcome variables

At each visit, participants were asked the frequency of their hot flashes and/or night sweats in the prior 2 weeks [24–26]. Prior analyses [2] indicated that these two symptoms were highly correlated, providing justification for examining them jointly. *Any* VMS was defined as reporting at least 1 day with hot flashes or night sweats within the prior 2 weeks, and *frequent* VMS as having VMS on 6 or more days in the prior 2 weeks [2].

#### Covariates

Time-invariant factors included race/ethnicity, site, visit 1 symptom sensitivity [27], menopausal stage and use of systemic menopausal hormone therapy (HT) at the index visit, baseline history of premenstrual symptoms, education, and ever receiving BrCa chemotherapy or radiotherapy (treatment timing was unavailable). Chemotherapy and radiotherapy were ascertained from interviews, medical records, and self-reported questionnaires from cases.

All other covariates were time-varying. Because current smoking [28] was stable, missing values were interpolated using last observation carried forward. Passive smoke exposure [29] and alcohol consumption were missing in more than 2700 observations and thus excluded; adjustment for these two characteristics yielded similar results in the subsample for whom they were available (data not shown). Psychosocial characteristics included elevated anxiety score [24] for the prior two weeks (score > 4 [30]) and elevated depressive symptom score (score ≥ 16 on the 20-item Center for Epidemiology Studies—Depression (CES-D) scale for the previous week [31]), both lagged one visit. Marital status was categorized as never, previously, or currently married/partnered. Self-assessed health was categorized as excellent, very good, good, and fair/poor. BMI was computed as weight in kg/(height in meters)^2^, categorized as < 25, 25–29.9, 30 + .

At all visits except 11 and 14, interviewers ascertained prescription medication use since the prior visit, transcribing preparation names from medication containers that participants brought into the visit. Medications analyzed here included HT, anti-anxiety medications, anti-depressants, BrCa SERMS (tamoxifen for all but one case), non-BrCa SERMs, and endocrine medications. BrCa treatment information (BrCa SERMS, endocrine medications [including aromatase inhibitors, GnRH agonists and (less frequently reported in our participants) estrogen receptor blockers and inhibitors of Type II 5α-reductase], chemotherapy and radiotherapy) was obtained in annual surveys and from a Pink SWAN questionnaire and medical records for women who reported incident BrCa. Medications were coded using the Iowa Drug Information Service system. Follow-up visits 11 and 14 were abbreviated; medications other than HT, employment, and self-reported health were not ascertained. Values were imputed using logistic or linear regression, based on adjacent visit values, age, menopausal status, race/ethnicity, site, and case/control status.

Menopausal status categories—both at the index visit and time-varying—included premenopausal (menses in the prior three months without change in menstrual regularity in the prior year), early peri-menopausal (menses in the prior 3 months and changes in regularity in the prior year), late peri-menopausal (3–11 months of amenorrhea) and naturally postmenopausal (≥ 12 months of amenorrhea). Women who had a hysterectomy and/or bilateral oophorectomy were grouped with naturally postmenopausal women at the index visit due to small numbers of such surgeries and similar rates of VMS in the two groups but were separated for time-varying menopausal status. Pre- and early peri-menopausal stages were combined due to the small number of premenopausal observations post diagnosis.

For women initiating menopausal HT or oral contraception use before their final menstrual period, menopausal status could not be discerned because menstrual bleeding was masked. If a woman stopped such hormone use and resumed bleeding, menopausal status was categorized according to the criteria above. Those who had the same status at both the visits prior to and following hormone use were categorized with that menopausal stage; otherwise menopausal status was considered missing.

### Data analyses

Participant characteristics were compared for cases and controls using chi-square or Fisher’s exact test for categorical variables and Student’s *t* test for continuous variables.

In addition to case/control status, the primary predictor was years before/after diagnosis/index date with diagnosis/index date = time 0. Diagnosis date was used as a surrogate for treatment initiation, which we assumed occurred shortly after diagnosis. Analyses of prevalent VMS omitted data outside the range − 10 to + 10 years to avoid sparse data.

Patterns of *prevalent* and *incident* VMS were examined in relation to the diagnosis/index date to distinguish patterns of persistent VMS in women who already had VMS from newly developed VMS, respectively. For both *prevalence* and *incidence, any* and *frequent* VMS were modeled separately.

*Prevalence of* VMS was modeled using random effects logistic regression [32]. Separately for cases and controls, we used nonparametric, locally weighted scatterplot smoothing (LOESS) regression by years before/after diagnosis/index [33]. Possible knots—points of inflection with a change in slope—suggested by LOESS were tested in piecewise linear modeling to identify the best-fitting knot placement. To allow cases and controls to have different slopes in segments delineated by knots, we included interactions of case/control status and segment-specific time variables in logistic regression models. Results from these models are presented as: (a) *point prevalences* at segment start and (b) segment-specific *per-year adjusted odds ratios* (aOR); the former indicates the *point prevalence* level at the start of segments, and the latter reflects change in odds with an additional 1 year elapsed.

To address hypothesis #1, in cases we compared point prevalence (PP) of VMS at diagnosis and post-diagnosis, and per-year aORs in different segments. For hypothesis #2, we compared PP and per-year aORs for cases versus controls in each segment. Covariate adjustment was performed in three stages: covariates other than BrCa treatments; adding medications (hypothesis #3); and adding chemotherapy and radiotherapy (hypothesis #3). For hypothesis #4, we tested interactions between case/control status and VMS risk factors previously identified in SWAN [2], stratifying on pre and post diagnosis/index. We also examined changes in HT use, as cases may have been more likely to stop HT post- diagnosis, potentially leading to “rebound” VMS.

*Incidence of* VMS was analyzed in participants without baseline VMS using Kaplan–Meier plots—and covariate-adjusted discrete survival analysis accounting for “late entry”—participants were excluded from risk sets earlier than their study entry [34].

All analyses used SAS 9.4 (SAS Institute Inc., Cary, NC, USA). *p* values < 0.05 were considered statistically significant.

## Results

Controls had a slightly but not statistically significantly higher *point prevalence* (PP) of VMS at baseline (39.3%) than cases (34.7%) (Table 1). Demographic, psychosocial and health characteristics did not differ between cases and controls, except cases tended to drink more alcohol (Table 1). Factors associated with VMS at baseline largely did not differ from those previously reported in SWAN [2] (data not shown).

**Table 1:** Comparison of baseline characteristics of cases and controls

	Controls (*N* = 2161)	Breast Cancer Cases (*N* = 151)	*p* value^a^
Race/ethnicity: *N* (%)			0.54
White	1045 (48.4)	75 (49.7)	
Black	598 (27.7)	44 (29.1)	
Chinese	196 (9.1)	10 (6.6)	
Japanese	216 (10.0)	18 (11.9)	
Hispanic	106 (4.9)	4 (2.6)	
Age in years: median, IQR^b^	46.3 (4.1)	46.6 (4.7)	0.26
Educational level: *N* (%)			0.36
No more than high school	457 (21.4)	27 (18.1)	
Some college	694 (32.3)	44 (29.5)	
College degree	996 (46.4)	78 (52.4)	
Marital status: *N* (%)			0.36
Currently married/living as married	1470 (68.8)	94 (63.5)	
Never married	263 (12.3)	23 (15.5)	
Previously married	403 (18.9)	31 (22.0)	
Difficulty paying for basics: *N* (%)			0.85
Not at all	143 (6.8)	10 (6.8)	
Somewhat	603 (28.5)	39 (26.4)	
Very	1372 (64.8)	99 (66.9)	
Current employment status: *N* (%)			0.93
No	393 (18.2)	27 (17.9)	
Yes	1768 (81.8)	124 (82.1)	
Self-reported health: *N* (%)			0.44
Excellent	493 (23.1)	34 (23.0)	
Very good	806 (37.7)	50 (33.8)	
Good	597 (27.9)	50 (33.8)	
Fair/poor	242 (11.3)	14 (9.5)	
Smoking: *N* (%)			0.44
Never	1281 (59.3)	91 (60.7)	
Past	577 (26.7)	34 (22.7)	
Current	302 (14.0)	25 (16.7)	
Passive smoking exposure: *N* (%)			0.88
None	1003 (46.7)	67 (44.7)	
1–4 person-h per week	587 (27.3)	42 (28.0)	
5 + person-h per week	558 (266.0)	41 (27.3)	
Body mass index, kg/m^2^: median, IQR	26.16 (8.9)	26.9 (9.6)	0.39
Alcohol use: *N* (%)			0.029
None	1022 (49.6)	61 (41.5)	
Light (< 1 drink/week)	201 (9.8)	19 (12.9)	
Moderate (1–7 drinks/week)	529 (25.7)	51 (34.7)	
Heavy (> 7 drinks/week)	309 (15.0)	16 (11.0)	
CES-D score ≥ 16: *N* (%)			0.79
No	1682 (77.9)	119 (78.8)	
Yes	478 (22.1)	32 (21.2)	
Visit 01 symptom sensitivity: median (IQR)	10.0 (4.0)	10.0 (5.0)	0.96
Anxiety (≥ 4 on scale): *N* (%)			0.76
No	1894 (87.8)	133 (88.7)	
Yes	263 (12.2)	17 (11.3)	
History of premenstrual symptoms: (*N*) %			0.90
No	241 (11.2)	17 (11.6)	
Yes	1905 (88.8)	130 (88.4)	
Menopause transition stage: *N* (%)			0.72
Premenopausal	1177 (54.5)	80 (53.0)	
Early peri-menopausal	984 (45.5)	71 (47.0)	
Vasomotor symptoms: *N* (%)			0.37
0 days in past 2 weeks	1307 (60.8)	98 (65.3)	
1–5 days in past 2 weeks	616 (28.7)	35 (23.3)	
6 + days in past 2 weeks	226 (10.5)	17 (11.3)	

^a^Chi-square test for categorical variables, Kruskall–Wallis test for continuous variables

^b^Interquartile range

**Fig. 2:**
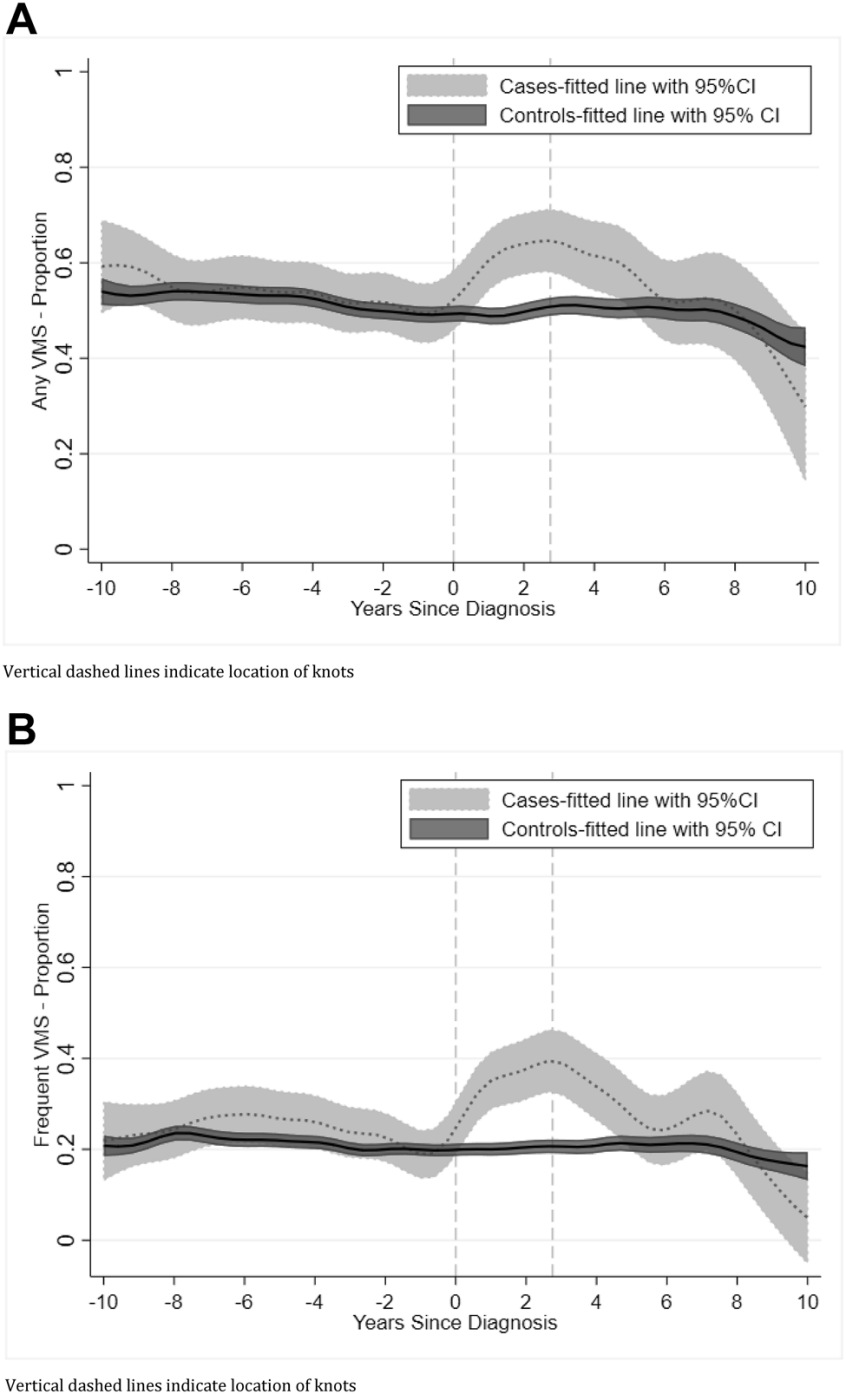
**a** Proportion *any prevalent* VMS, loess curve over time prior to and after diagnosis/index date for cases and controls. **b** Proportion *Frequent Prevalent* VMS, loess curve over time prior to and after diagnosis/index date for cases and controls

The LOESS plot of the PP of *any* VMS among cases (hypothesis #1) suggested two knots with an increase starting at diagnosis (year 0) and a decrease starting at 2.75 years post-diagnosis (Fig. 2a). Among controls, VMS decreased gradually over time (hypothesis #2). Patterns were similar for the PP for *frequent* VMS (Fig. 2b). Consequently, all models used two knots, estimating separate per-year aORs for cases and controls in each segment: Segment 1 = up to 10 years pre-diagnosis/index; Segment 2 = diagnosis/index to 2.75 years post-diagnosis/index; and Segment 3 = 2.75–10 years post-diagnosis/index.

For hypothesis #1, analyses only in cases, adjusted PP of *any* VMS declined slightly pre-diagnosis (per-year aOR = 0.91; Table 2, first model), but increased sharply in Segment 2 with PP at 2.75 years post diagnosis was almost twice the PP at diagnosis (77.9% versus 42.6%, *p* < 0.001). The per-year aOR for cases in Segment 2 is 1.76, indicating that for each additional passing year, the odds of any VMS increased by a factor of 1.76. Subsequently, prevalence rates declined (Segment 3 per-year aOR = 0.72). Pairwise differences between segment-specific per-year aORs were statistically significant (*p* < 0.004).

**Table 2:** Multivariable random effects binomial logistic regression for prevalence of any VMS from 10 years pre- to 10 years post-diagnosis, Pink SWAN

	Segment 1: -10 years to diagnosis/index		Segment 2: diagnosis/index to 2.75 years post diagnosis/Index		Segment 3: 2.75–10 years post diagnosis/index	
	Adjusted % point prevalence (SE) at -10 years	Per-year odds ratio^a^ (95% CI)	Adjusted % point prevalence (SE) at diagnosis/index	Per-year odds ratio^a^ (95% CI)	Adjusted % point prevalence (SE) at + 2.75 years	Per-year odds ratio^a^ (95% CI)
Adjusted^b^						
Controls	51.0 (3.3)	0.97 (0.94, 0.99)	42.3 (2.2)	1.04 (0.99, 1.11)	45.2 (2.7)	0.96 (0.92, 1.00)
Breast cancer cases	65.4 (7.2)	0.91 (0.84, 0.98)	42.6 (6.2)	1.76 (1.39, 2.24)	77.9 (5.4)	0.72 (0.61, 0.84)
*p* Values, case–control difference^c^	0.053	0.123	0.964	< 0.001	< 0.001	< 0.001
Additional adjustment for time-varying non-BrCa SERM, BrCa SERM, and BrCa endocrine medications						
Controls	51.1 (3.3)	0.97 (0.94, 0.99)	42.4 (2.2)	1.04 (0.98, 1.11)	45.2 (2.7)	0.96 (0.92, 1.00)
Breast cancer cases	65.3 (7.2)	0.91 (0.84, 0.98)	41.7 (6.2)	1.56 (1.20, 2.04)	71.0 (7.5)	0.77 (0.65, 0.91)
*p* Values, case–control difference^c^	0.057	0.107	0.916	0.0034	0.0026	0.0093
Further adjustment for ever chemo- and ever radiotherapy						
Controls	51.2 (3.3)	0.96 (0.94, 0.99)	42.2 (2.2)	1.04 (0.98,1.11)	45.0 (2.7)	0.96 (0.92, 1.00)
Breast cancer cases	53.1 (11.2)	0.91 (0.84, 0.98)	30.2 (8.7)	1.63 (1.23, 2.16)	62.5 (11.5)	0.76 (0.64, 0.91)
*p* Values, case–control difference^c^	0.87	0.128	0.206	0.002	0.141	0.010

^a^Per-year odds ratio reflects the change in odds associated with a 1-year increase in that segment

^b^Adjusted for: menopause status and hormone use at first post-diagnosis visit, site, race/ethnicity and time-varying menopause transition stage and hormone use, age, body mass index, education, self-reported health, marital status, employment status, symptom sensitivity at visit 01, 1-year lagged anxiety score > 4, 1-year lagged anxiety medication use, and use of anti-depressants, history of premenstrual symptoms and smoking status

^c^For prevalences at start of segments, *p* value indicates statistical significance for the case–control difference in VMS prevalence. For per-year ORs, the *p* value indicates statistical significance for the case–control difference in the time slope, i.e., for the interaction between case–control status and segment time

In hypothesis #2 analyses, adjusted PP of *any* VMS was somewhat higher in cases than controls pre-diagnosis/index (*p* = 0.053) (Table 2, first model), but decreased similarly for both groups in Segment 1 (*p* = 0.12). In Segment 2, in contrast to the sharp increase in PP noted above in cases, PP in controls was stable (per-year aOR = 1.04, *p* < 0.001 for case/control difference), leading to a significantly higher PP at 2.75 years post-diagnosis/index for cases than controls (77.9% vs. 45.2%, *p* < 0.001). Conversely, in Segment 3, VMS PP declined significantly in cases as noted above but remained stable in controls (per-year aOR 0.96; *p* < 0.001 for case/control difference). Adjusted PPs for *frequent* VMS were lower (6.7–9.5% among controls, 7.6–27.1% among cases), but case/control differences in PP and per-year aORs were consistent with those for *any* VMS (data not shown).

New HT use at the first post-diagnosis/index visit was low in both cases and controls (3.9% versus 4.3%, respectively, *p* = 0.85). However, continued use among cases was significantly lower than among controls at the first post-diagnosis visit (28.6% versus 61.8%, *p* = 0.003). Adjustment for HT patterns had a negligible effect on per-year aORs, however (data not shown), nor was the interaction of HT pattern with case/control status statistically significant (*p* = 0.67).

Use of BrCa SERMS occurred almost entirely among cases post-diagnosis (29.0% of Segment 2 observations, 14.4% of Segment 3 observations), with only two Segment 1 observations (0.01%) in controls. Endocrine medication use was reported in 15.6% and 18.2% of cases’ Segment 2 and Segment 3 observations, respectively, versus 0.10% and 0.03% among controls. In hypothesis #3 analyses, concurrent BrCa SERM use was positively associated with prevalence of *any* VMS (aOR = 2.95, 95% CI 1.34, 6.52), but concurrent endocrine medication use was unrelated (aOR = 0.96, 95% CI 0.46, 1.97). The latter result may reflect endocrine medication use only during postmenopause, rather than in late peri-menopause when VMS PP tends to peak [2]. Adjustment for medications reduced the Segment 2 per-year aOR for *any* VMS among cases from 1.76 to 1.56 (Table 2, second model), but it remained significantly higher than for controls (*p* = 0.003). The pattern was similar for prevalence of *frequent* VMS (data not shown).

Among cases, 41.3% received chemotherapy, and 70.9% received radiotherapy. Neither was significantly associated with the prevalence of *any* VMS (chemotherapy aOR = 0.96, 95% CI 0.43, 2.15; radiotherapy aOR = 1.86, 95% CI 0.79, 4.37), nor did adjustment affect case/control differences in PPs of VMS and per-year aORs (Table 2, third model).

**Fig. 3:**
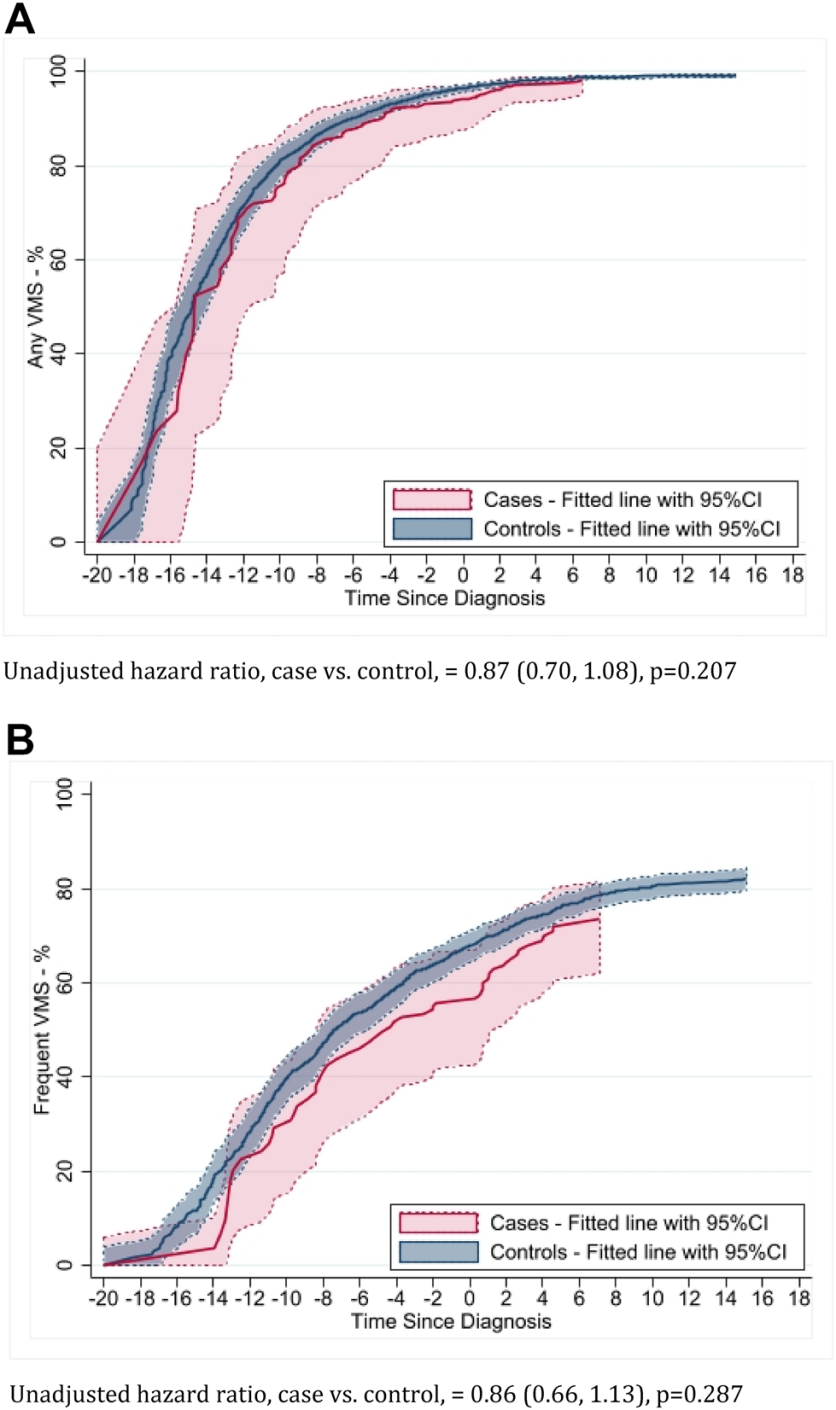
**a** Kaplan–Meier Plot of cumulative probability (with 95% Confidence Intervals) of any incident VMS over time prior to and after diagnosis/index date for cases and controls. **b** Kaplan–Meier plot of cumulative probability (with 95% Confidence Intervals) of frequent incident VMS over time prior to and after diagnosis/index date for cases and controls

Similar to previous SWAN findings [22], Kaplan–Meier plots suggested slightly lower incidence of *any* VMS (Fig. 3a) and *frequent* VMS (Figs. 3b) in cases than controls with almost all incidence for both groups occurring pre-diagnosis/index. This resulted in small numbers of *incident* VMS post-diagnosis/index (19–21 incidents in cases), making multivariate analyses of these trends unstable. Overall, aHR (95% CI) for cases versus controls were 1.16 (0.79, 1.70) for *any* VMS and 1.10 (0.74, 1.63) for *frequent V*MS. Among participants with no VMS pre-diagnosis, corresponding aHRs post-diagnosis/index were 1.37 (0.61, 3.07) and 1.55 (0.84, 2.87), respectively.

In hypothesis #4 analyses, no statistically significant case/control interactions with risk factors were observed for prevalence of *frequent* VMS, either pre- or post-diagnosis/index. For prevalence of *any* VMS, two interactions were statistically significant: race/ethnicity pre-diagnosis (interaction *p* = 0.043) and menopause status post-diagnosis (interaction *p* = 0.026): aORs for Black versus white were 9.04 for cases versus 2.09 for controls, and for Hispanic versus white were 24.4 for cases versus 0.52 for controls; aORs for late versus early peri-menopause were 13.0 for cases but 3.77 for controls; aORs for post- versus early peri-menopause were 12.5 for cases but 2.80 for controls. Given the large number of comparisons and small cell counts (7 Hispanic observations, 33 late peri-menopause observations, and 36 pre-/early peri-menopause in cases), these significant interactions may have been due to chance.

## Discussion

Our results provide a new contribution by comparing pre- and particularly post-diagnosis trajectories of VMS among women with incident BrCa to those of controls, revealing that much of the increase post-diagnosis was not attributable to treatment, and revealed that VMS risk factors did not differ in cases and controls.

*Point prevalence* of *any* VMS was similar for both groups pre-diagnosis/index, consistent with prior SWAN findings [20]. This differed from findings from a nearly 18-year follow-up of 25,499 postmenopausal non-HT users in the Women’s Health Initiative, which revealed that women with VMS lasting 10 + years had higher BrCa incidence than those who never reported VMS [35]. In our study, the PP of VMS increased in cases from diagnosis to + 2.75 years but remained stable among controls, suggesting treatment effects in cases, supported by the significance of the BrCa SERMs variable, consistent with prior findings [21]. However, adjustment for BrCa SERM treatment only somewhat attenuated the still significantly elevated per-year aOR among cases, suggesting that treatment only partially accounted for the VMS increase in contrast to findings from prior studies of BrCa cases taking aromatase inhibitors [6, 7] or tamoxifen [8]. Consistent with prior results [3], chemotherapy had no additional impact on VMS prevalence, which declined in cases and controls after 2.75 years after diagnosis/index with a steeper decline among cases.

Most *incident* VMS in both groups occurred pre-diagnosis/index, resulting in small numbers of *incident* VMS post diagnosis/index; thus, *incidence* trends were unstable, but indicated no statistically significant case/control differences in post-diagnosis/index *incident* VMS. Because analyses of *prevalent* VMS included women who had VMS in prior segments, but analyses of *incident* VMS did not, the higher per-year aOR for prevalence of VMS among cases (after adjustment for BrCa SERMs), but not *incidence* of VMS in Segment 2 could reflect a predisposition for VMS among cases or common underlying factors (eg, inflammation) for BrCa [36] and VMS [37].

Our study’s strengths included: (a) the longitudinal design, permitting assessment of temporal relations of VMS, BrCa and VMS risk factors; (b) inclusion of five racial/ethnic groups, providing good generalizability; and (c) standardized data, enabling statistical adjustment for many potential covariates, reducing residual confounding. The study had limitations, however. First, most notably, the number of incident BrCa cases was relatively small, potentially resulting in some modest but meaningful associations not detected as statistically significant, such as when examining *incident* VMS and case/control status interaction with risk factors. Second, recall errors and misclassification could have resulted from self-reported data on VMS, BrCa treatments, and medications; however, we verified reported medications against containers for prescribed medications that women brought to each visit. Third, we lacked information regarding timing of chemotherapy or radiotherapy so that we could not examine concurrent treatment but only any vs. no treatment, which may explain the lack of significant associations. Finally, most cases occurred postmenopausally so that the menopause-inducing effects, including VMS, of some BrCa therapies reported in prior studies [9, 10] were not observable.

In conclusion, risk factors for VMS did not differ between cases and controls, but trajectories of *prevalence of* VMS differed significantly. Prevalence of VMS remained stable among controls, but increased in cases in the immediate post-diagnosis segment, partially reflecting treatment but also possibly reflecting common underlying factors for BrCa and VMS or a predisposition for VMS among cases. However, *incidence of* VMS did not increase post- diagnosis, arguing against predisposition, but not against common factors for VMS and BrCa. These findings suggest that *prevalence of* VMS post-diagnosis was only partially explained by treatment and possibly by prior *prevalence of* VMS in cases because analyses of VMS *prevalence* included women who had VMS previously, again suggesting shared underlying factors for VMS and BrCa. That post-diagnosis VMS were not entirely attributable to treatments which is important clinically because VMS could lead to lower adherence to BrCa treatment. Additionally, we did not find higher HT use in cases than controls pre-diagnosis so that higher prevalence of VMS soon after BrCa diagnosis does not seem attributable to prior HT use.

## Data Availability

Public use datasets for SWAN data are available at the AgingResearchBiobank https://agingresearchbiobank.nia.nih.gov/studies/swan/. The breast cancer data underlying this article were obtained through a separate source of funding (1 R01 CA199137-01) and are not shared publicly because of confidentiality. The data will be shared on reasonable request to the SWAN Coordinating Center, with review by the PI of that funding, Dr. Nancy Avis, and will require a formal data use agreement between the applicant’s institution and the University of Pittsburgh.

## Code availability

Code for the analyses for this paper will be shared on reasonable request to the SWAN Coordinating Center and will require a formal use agreement between the applicant’s institution and the University of Pittsburgh.
